# Gut dysbiosis and clinical phases of pancolitis in patients with ulcerative colitis

**DOI:** 10.1002/mbo3.1181

**Published:** 2021-05-01

**Authors:** Brenda Maldonado‐Arriaga, Sergio Sandoval‐Jiménez, Juan Rodríguez‐Silverio, Sofía Lizeth Alcaráz‐Estrada, Tomás Cortés‐Espinosa, Rebeca Pérez‐Cabeza de Vaca, Cuauhtémoc Licona‐Cassani, July Stephany Gámez‐Valdez, Jonathan Shaw, Paul Mondragón‐Terán, Cecilia Hernández‐Cortez, Juan Antonio Suárez‐Cuenca, Graciela Castro‐Escarpulli

**Affiliations:** ^1^ Laboratorio de Metabolismo Experimental e Investigación Clínica División de Investigación Clínica C.M.N. “20 de Noviembre” ISSSTE and Hospital General de 2A Troncoso Instituto Mexicano del Seguro Social Ciudad de México México; ^2^ Laboratorio de Investigación Clínica y Ambiental Departamento de Microbiología Escuela Nacional de Ciencias Biológicas Instituto Politécnico Nacional Ciudad de México México; ^3^ Escuela Superior de Medicina Instituto Politécnico Nacional Ciudad de México México; ^4^ Unidad de Análisis y Referencia Virológica C.M.N. "20 de Noviembre" ISSSTE Ciudad de México México; ^5^ Clínica de Enfermedad Inflamatoria Intestinal Servicio de Gastroenterología C.M.N. “20 de Noviembre” ISSSTE Ciudad de México México; ^6^ Coordinación de Investigación y División de Investigación Biomédica C.M.N. “20 de Noviembre” ISSSTE Ciudad de México México; ^7^ Laboratorio de Genómica Industrial Centro de Biotecnología FEMSA Tecnológico de Monterrey Monterrey NL Mexico; ^8^ Department of Infection, Immunity & Cardiovascular Disease University of Sheffield Medical School Sheffield UK; ^9^ Laboratorio de Bioquímica Microbiana Departamento de Microbiología Escuela Nacional de Ciencias Biológicas Instituto Politécnico Nacional Ciudad de México México

**Keywords:** active and remission phase, gut dysbiosis, gut microbiota, pancolitis

## Abstract

Ulcerative colitis (UC) is a frequent type of inflammatory bowel disease, characterized by periods of remission and exacerbation. Gut dysbiosis may influence pathophysiology and clinical response in UC. The purpose of this study was to evaluate whether gut microbiota is related to the active and remission phases of pancolitis in patients with UC as well as in healthy participants. Fecal samples were obtained from 18 patients with UC and clinical‐endoscopic evidenced pancolitis (active phase *n* = 9 and remission phase *n* = 9), as well as 15 healthy participants. After fecal DNA extraction, the 16S rRNA gene was amplified and sequenced (Illumina MiSeq), operational taxonomic units were analyzed with the QIIME software. Gut microbiota composition revealed a higher abundance of the phyla *Proteobacteria* and *Fusobacteria* in active pancolitis, as compared with remission and healthy participants. Likewise, a marked abundance of the genus *Bilophila* and *Fusobacteria* were present in active pancolitis, whereas a higher abundance of *Faecalibacterium* characterized both remission and healthy participants. LEfSe analysis showed that the genus *Roseburia* and *Faecalibacterium* were enriched in remission pancolitis, and genera *Bilophila* and *Fusobacterium* were enriched in active pancolitis. The relative abundance of *Fecalibacterium* and *Roseburia* showed a higher correlation with fecal calprotectin, while *Bilophila* and *Fusobacterium* showed AUCs (area under the curve) of 0.917 and 0.988 for active vs. remission pancolitis. The results of our study highlight the relation of gut dysbiosis with clinically relevant phases of pancolitis in patients with UC. Particularly, *Fecalibacterium*, *Roseburia*, *Bilophila*, and *Fusobacterium* were identified as genera highly related to the different clinical phases of pancolitis.

## Graphical abstract

The purpose of this study was to evaluate whether gut microbiota is related to the active and remission phases of pancolitis in patients with ulcerative colitis, as well as in healthy participants.

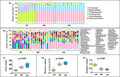

## 1 INTRODUCTION

The intestinal tract houses a large and diverse community of microorganisms collectively referred to as the gut microbiota. These microorganisms contribute to human health by promoting both immune and metabolic functions (Burman et al., 2016; Chassaing et al., 2017). It is widely accepted that the gut microbiota has a crucial role in regulating the function of the intestinal epithelium, the immune system, and its homeostasis within the gut (Imhann et al., 2018). The term “dysbiosis” refers to an imbalance in the composition and function of the microbiota (Danilova et al., 2019; Nishida et al., 2017; Vemuri et al., 2017), whereas gut dysbiosis along with the altered host immune response has been observed in clinically relevant immunological and inflammatory diseases, such as Ulcerative Colitis (UC), which is a frequent type of Inflammatory Bowel Disease, characterized by periods of remission and exacerbation (Nishida et al., 2018). UC has been classified according to its extent and severity in the so‐called Montreal classification, defining the extent as E1 indicates ulcerative proctitis; E2 as UC on the left side; and E3 as extensive UC or pancolitis. Likewise, the severity of the disease is classified into clinical remission (S0), mild disease (S1), moderate disease (S2), and severe disease (S3). Pancolitis is considered the most serious clinical phase of UC, and its involvement represents 10%–15% of all UC (Mohammed Vashist et al., 2018).

Although a causal effect has not been evidenced; nowadays, it is widely accepted that altered interactions between gut dysbiosis and the intestinal immune system promote UC (Imhann et al., 2018), while the precise nature of the intestinal microbiota dysfunction in UC remains to be elucidated. In this sense, the gut microbiota has been considered as a “fingerprint” reflecting the natural history of UC, since it associates with the clinical severity, remission, and flare‐up responses (Marchesi et al., 2016). Gut microbiota from patients with UC has been characterized by a reduced number of bacteria with anti‐inflammatory capacities and a higher proportion of bacteria with pro‐inflammatory properties. Microbiota diversity is also reduced; low abundance of microorganisms like *Firmicutes* and high abundance of *Proteobacteria* have been found (Manichanh et al., 2012; Yu, 2018). Rapid development and application of culture‐independent, high throughput DNA‐based sequencing technologies have elicited the recognition of such dysbiotic signatures, which may play a role during the early identification of clinical‐therapeutic phases of UC, and particularly useful in severe clinical manifestations like pancolitis (Peterson et al., 2008; Rintala et al., 2017). Despite this notion, the relation of gut dysbiosis with pancolitis has been poorly characterized. Given the increasing UC prevalence worldwide, including Latin American countries (Bosques‐Padilla et al., 2011; Farrukh & Mayberry, 2014), along with the strong interest to understand the relation of dysbiotic gut microbiota with most serious phases of UC like pancolitis, the present study aimed to characterize gut microbiota from patients with UC and different clinical phases of pancolitis.

## 2 METHODS

### 2.1 Study population

In this cross‐sectional study, groups of 18 patients with UC and clinical‐endoscopic‐evidenced pancolitis (active phase *n* = 9 and remission phase *n* = 9) as well as 15 healthy participants, attended the Department of Gastroenterology, *Centro* *Médico* *Nacional* *‘20* *de* *Noviembre’* ISSSTE, Mexico City, Mexico, between July 2017 and January 2019. Patients with concomitant irritable bowel syndrome, pseudomembranous colitis, and antibiotic treatment during the previous 4 weeks were excluded. Pancolitis was defined according to clinical, radiological, endoscopic, and histological criteria (Van Assche et al., 2010). All the patients had experienced at least one previous episode of pancolitis before their recruitment. The study at remission phase of pancolitis received therapy based on pharmacological treatment, a fiber‐rich diet, and the use of probiotics (Owczarek et al., 2016). Some patients with active pancolitis did not receive treatment due to non‐medical reasons, like the inability to attend their follow‐up appointment. Characteristics like age, time since disease onset, affected gastrointestinal location, frequency of bowel movements, and presence of blood in stools were collected from clinical records. Clinical activity was defined as a value of 4 or higher for colitis activity index (CAI [Clinical activity index], used for ulcerative colitis), and clinical remission was defined with CAI value <2 for at least 3 months (Siegel et al., 2018; Van Assche et al., 2010). Healthy participants were volunteers without previous history of chronic disease, belonging to a different family than those with UC, but with a similar diet, as assessed by a 24‐h recall (R24H) survey (Parks et al., 2018).

### 2.2 Stool samples

Stool samples were collected either during hospitalization (active pancolitis) or prepared at home and collected during programmed medical consultation (remission phase and healthy participants); samples were stored at home between 4 and 8°C for up to 24 h, before hospital collection. Samples were collected with the help of a stool sampling kit, which consisted of a plastic lining to cover the toilet, two stool sample tubes with spoons, two plastic bags, and a clipping system for safe closure of the outer bag. Samples were labeled upon arrival, and one part was processed for fecal calprotectin assay; while the remaining was aliquoted and frozen directly at −80°C for further microbiota analyses (Tedjo et al., 2015).

### 2.3 DNA extraction of fecal samples

Frozen stool samples were thawed on ice, and approximately 200 mg were added to dry‐bead tubes with lysis buffer (AllPrep PowerFecal DNA, Qiagen). The stool samples were homogenized followed by a combined chemical and mechanical lysis by using prefilled lysis tubes. Inhibitors commonly present in stool samples were then removed before isolation of nucleic acids. DNA isolation was continued by using the AllPrep DNA MiniElute spin column, according to the manufacturer's instructions. DNA was eluted in 30 μl EB‐buffer. Negative control samples (consisted only of PCR grade water) were handled in the same way as the fecal samples, to rule out contamination during the isolation procedure (Tedjo et al., 2015). A Nanodrop ND‐1000 (NanoDrop Technologies), was used to estimate DNA concentrations. DNA concentration was adjusted to a final concentration of 10 ng/µl (Tedjo et al., 2016).

### 2.4 Amplification and sequencing of bacterial 16S rRNA gene

The V3 and V6 hypervariable regions of the *16S* *rRNA* gene were PCR amplified from microbial genomic DNA with the forward (TAT GGT AAT TGT GTG CCA GCM GCC GCG GTA A) and reverse (GGA CTA CHV GGG TWT CTA AT) primers. The primers were designed with overhanging adapters (Forward: AATGATA CGGC GACC ACCGA GATCT ACAC), (Reverse: GGA CTA CHV GGG TWT CTA AT) for annealing to Illumina universal index sequencing adaptors that were added in a later PCR (Dubinsky & Braun, 2015; Haas et al., 2011). The PCR products were evaluated by 2% agarose gel electrophoresis and purified. After purification, spectrophotometry was used to quantify the PCR products. Samples were normalized to a final concentration of 2 nM.

### 2.5 Microbial composition and analysis by Illumina

A two‐steps PCR methodology was used to prepare *16S* *rRNA* libraries. For the first‐step, extracted DNA was quantified and samples were diluted to the amount of the least concentrated sample. Then 2 μL were used for the PCR reaction (quadruplicates) at the following conditions: 98°C for 30 s [98°C for 30 s, 52°C for 30 s, 72°C for 30 s] for 20 cycles, 4°C hold. Then, the 4 resulting reactions were amalgamated. The samples were then cleaned by using AmpureXP beads and eluted in 40 μL final volume. For the second step, a 4 μL of the obtained DNA was mixed with primers PE‐PCR‐III‐F and PE‐PCR‐IV‐barcode, in a 25 μL final volume PCR reaction (quadruplicates), at run cycle conditions of 98°C for 30 s [98°C for 30 s, 83°C for 30 s, 72°C for 30 s] for 7 cycles, 4°C hold. Then, the 4 PCR reactions were pooled and the products were cleaned by using *16S* *Metagenomic* *Sequencing* *Purification* beads (Caporaso et al., 2012). The DNA library concentrations were quantified and then multiplexed to provide the same amount of DNA in each sample. A single *Illumina* *MiSeq* lane set for paired‐end 300‐basepair reads was used to sequence the libraries. Paired‐end reads of *16S* *rRNA* gene libraries were generated with the Illumina, *MiSeq* platform. A total of 10,629,314 raw sequences were obtained, with further quality filter and binned resulting in 8,349,697 usable sequences, with a sample average of 378,489 per sequence. Sequences were clustered and singletons removed; the data were rarefied to control for variations in sequencing efforts. The datasets supporting the conclusions of this article are available in https://www.ncbi.nlm.nih.gov/bioproject/596546, under the ID PRJNA596549 repository. The analyses of taxonomy and diversity of the samples were performed taking as a reference the SILVA database (Bokulich et al., 2013; Bolyen et al., 2018).

### 2.6 Bioinformatic analysis

The Illumina Real‐Time Analysis software (version 1.17.28) was used for base calling, image analysis, and error estimation. Sequencing provided read lengths of 300 bp, which were demultiplexed, verifying that the paired ends provided a clear overlap. The paired ends were then linked together with the fastq‐join program (http://code.google.com/p/ea‐utils/). Separate files of each sample (R1 and R2) were entered in fastq format by using the split_libraries_fastq.py pipelines. Sequences that had quality value (QV) scores of ≥20 (Phred score of 20) for no‐less than 99% of the sequence were selected for further study. All sequences with ambiguous base calls were discarded. Subsequently, the sequences were grouped in Operational Taxonomic Units (OTU), where the pick_closed_reference_otus.py pipelines were used. QIIME, which uses the BIOM format, was used to represent OTU tables (Bolyen et al., 2018; Dubinsky & Braun, 2015; Edgar et al., 2011). Analyses of sequence reads were performed by using SILVA multiclassifier tools with a 97% confidence threshold (Navas‐Molina et al., 2013). Subsequent analyses of diversity index were all performed based on this output normalized data (Allali et al., 2017; Aßhauer et al., 2015). To perform the diversity analyses, the core_diversity_analyses.py pipelines were executed with the pipeline alpha_diversity.py. Alpha diversity metrics were calculated with QIIME, that is, the observed OTUs (observed species) and the phylogenetic diversity or complete tree PD (PD_whole_tree) (Bolyen et al., 2018); whereas the weighted distances of UniFrac of the beta diversity were determined with beta_diversity.py pipelines, and the R software v.2.15.3 was used to display the results (Barwell et al., 2015; Chao et al., 2006; Hass et al., 2011). The “Linear discriminant analysis (LDA) effect size (LEfSe)” algorithm was performed with the Galaxy online platform to determine the different relative abundances of bacterial communities among the different groups of patients. The significance thresholds used were those recommended in the program. LEfSe considered statistical significance between the different biological classes with a Kruskal–Wallis test and subsequently analyzed the biological significance with a Wilcoxon test (Segata et al., 2011).

### 2.7 Fecal calprotectin test

Fecal calprotectin (FC) was measured as a marker of intestinal inflammation by using a commercial ELISA (MyBioSource), following the manufacturer's instructions. Optical densities were read at 405 nm with a microplate ELISA reader. Samples were tested in duplicate, and results were calculated from a standard curve and expressed as μg/g stool (Chang & Cheon, 2018).

### 2.8 Statistical analysis

Data normality was evaluated with the Shapiro–Wilk test. Quantitative data were compared by non‐paired, two‐tail, *t* test, or *U*‐Mann Whitney, as appropriate. Analyses of the sequences were carried out in the QIIME and R software. Multivariate nonparametric ANOVA was used to determine the differences in the abundance of the microbial community between groups, whereas Unifrac was used to compare the abundance of the specific microbiota and the concentration of fecal calprotectin, and it was visualized by principal coordinate analysis. To test whether the clusters of microbiota from the study conditions were different between them, Unifrac *p*‐values, based on principal coordinate analysis applied to the matrix distance, were performed to allow pairwise comparison of microbiota from clinical phases of pancolitis and healthy controls (Caporaso et al., 2010; Lawley & Tannock, 2017). Finally, the Area Under the Curve (AUC) was calculated to explore whether the relative abundance of the bacterial genus most frequently observed (cutoff value according to ROC analysis) may predict UC severity. The Statistical Package for Social Sciences SPSS v.18.0. was used, and *p*‐values of ≤0.05 (2‐tailed) were considered to be statistically significant.

## 3 RESULTS

### 3.1 Study population

Eighteen patients diagnosed with UC and pancolitis, mean aged 37‐years‐old constituted the study population, who were further divided according to the disease activity, as demonstrated by the CAI and fecal calprotectin values. A cohort of sex‐ and age‐matched, healthy volunteers were included for comparison. Baseline clinical‐demographic characteristics are shown in Table 1. Stools from patients with active pancolitis were characterized by being watery, corresponding to Bristol type 7, and bloody (two points in rectal bleeding of Mayo Clinical Score) in all cases.

**TABLE 1:** Demographic and clinical characteristics of the study population (*n* = 33)

	AP (*n* = 9)	RP (*n* = 9)	HS (*n* = 15)	*p*‐value
Age (years old)	36.9 ± 1.4	37.9 ± 1.1	36. 4 ± 1.6	NS
Male	7 (77.7)	6 (66.6)	6 (40)	NS
Index CAI	11.0 ± 1.3	1.7 ± 0.6	N/A	<0.05
Montreal A (age at onset)				
A1 (16)	None	None	N/A	NS
A2 (17–40)	7 (77.7)	6 (66.6)		
A3 (41)	2 (22.2)	3 (33.3)		
Montreal Score Extensive				
E1 ulcerative proctitis	None	None	N/A	NS
E2 left‐sided UC	None	None		
E3 extensive UC	9 (100)	9 (100)		
Montreal Score Severity				
S0 silent colitis	None	9 (100)	N/A	NS
S1 mild colitis	None	None		
S2 moderate colitis	None	None		
S3 severe colitis	9 (100)	None		
Endoscopy Mayo Score				
0	NONE	N/A	N/A	N/A
1	NONE			
2	NONE			
3	9 (100)			
Frequency of bowel movements	≥ 10	2 to 4	1 to 2	NS
Presence of blood in stools	9 (100)	None	None	NS
Time (years) from diagnosis				
≥10	8 (88.8)	6 (66.6)	N/A	NS
≤10	1 (11.1)	3 (33.3)		
Currently smoking	2 (22.2)	None	None	N/A
Medication use				
Mesalazine	None	6 (66.6)	N/A	NS
Corticosteroids	2 (22.2)	2 (22.2)		
Infliximab	none	1 (11.1)		
No treatment	7 (77.7)	None		
Fecal calprotectin (μg/g)	480.1 ± 13.7	99.6 ± 8.9	21.6 ± 4.3	*p* < 0.05

Quantitative data were resumed as mean ± SD and qualitative data as *n* (%). Statistical analysis was performed with a two‐way *U‐*Mann Whitney and Fisher's test, as appropriate.

Abbreviations: AP, active pancolitis; HS, healthy subjects; N/A, not applicable; NS, non‐significant; RP Remission pancolitis.

### 3.2 Microbial composition and diversity

**FIGURE 1:**
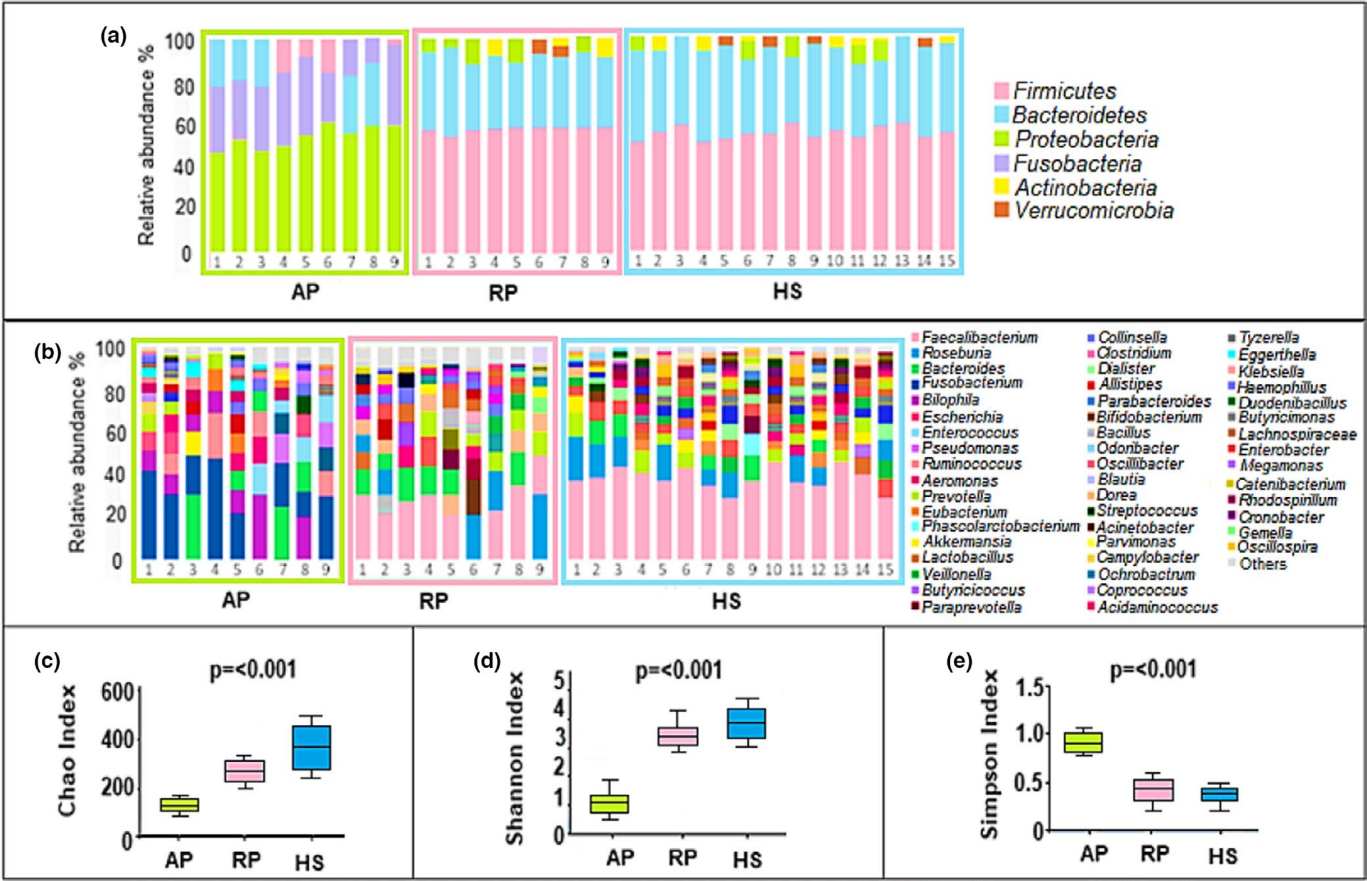
Characteristics of the microbial community in pancolitis, remission, and healthy participants. (a) Taxonomic composition distribution in samples of phylum level. (b) The taxonomic composition distribution in samples of genus level. (c) Alpha diversity index boxplot, including community richness (Chao), (d) diversity (Shannon), and (e) Dominance (Simpson). the *p*‐value indicates the statistical significance of two‐way  ANOVA. Abbreviations: AP, active pancolitis; HS, healthy subjects; RP, Remission pancolitis

The analysis of microbiome from fecal samples showed the relative abundance of OTUs at different taxonomic levels (Figure 1a, b, Table 2). OTUs were created out of the filtered tags and were grouped at a similarity of 97%. This gave a total of 1533 OTUs for the 33 samples used in this study. Taxonomic composition at the level of phyla is summarized in Figure 1a. The bacterial phyla *Firmicutes*, *Bacteroidetes*, *Proteobacteria*, and *Fusobacteria* were the most common sequences showing 97% of similarity. For remission pancolitis and healthy participants, *Firmicutes* was the most abundant bacterial phylum. Microbiota abundance in remission pancolitis was very similar to that observed in healthy participants, at the phyla level; whereas, active pancolitis showed phylum *Proteobacteria* as the most abundant. Genus distribution provided a subjective perception of the difference between the relative abundance of patients with active vs. remission pancolitis and healthy participants (Figure 1b) The most abundant genera in active pancolitis were *Fusobacterium* and *Bilophila*. For the group of remission pancolitis and healthy participants, the most abundant genera were *Faecalibacterium*, *Roseburia*, and *Bacteroides* (Appendix: Table A1, A2, and A3). Regarding bacterial alpha diversity comparison, pancolitis activity was related to the lowest community richness (Chao index) and diversity (Shannon index) (Figure 1c, d), whereas community richness and diversity were similar between remission pancolitis and healthy participants. Likewise, significant differences in species dominance of microbiota (Simpson index) (Figure 1e) were found between active vs. remission pancolitis and healthy participants (Appendix: Table A4).

**TABLE 2:** Comparison of gut dysbiosis in fecal samples from the pancolitis population

	Most abundant gut microbiota	AP (*n* = 9)	RP (*n* = 9)	HS (*n* = 15)
Phylum	*Firmicutes*	4.0 ± 1.5*/**	50.0 ± 5.2	54.6 ± 6.4
	*Bacteroidetes*	15.0 ± 0.2*/**	46.0 ± 4.2	45.0 ± 3.4
	*Proteobacteria*	52.5 ± 5.6*/**	0.0 ± 0.0	2.5 ± 1.0
	*Fusobacteria*	30.0 ± 2.5*/**	0.0 ± 0.0	0.0 ± 0.0
	*Actinobacteria*	1.5 ± 0.5	2.5 ± 1.0	2.5 ± 1.0
	*Verrucomicrobia*	0.0 ± 0.0	1.5 ± 0.3	1.5 ± 0.5
Genus	*Lactobacillus*	0.0 ± 0.0*/**	5.6 ± 4.2	8.5 ± 2.4
	*Faecalibacterium*	0.5 ± 1.5*/**	21.0 ± 8.7***	40.2 ± 4.9
	*Roseburia*	0.0 ± 0.0*/**	5.4 ± 7.2***	7.3 ± 7.4
	*Bacteroides*	7.6 ± 4.1	11.5 ± 10.8***	3.5 ± 2.1
	*Bilophila*	12.0 ± 9.1*/**	0.0 ± 0.0	0.0 ± 0.0
	*Fusobacterium*	35.6 ± 15.4*/**	0.0 ± 0.0	0.0 ± 0.0

Relative abundance is shown as mean ± SD and (†) percentage of the relative abundance in relation to that observed in healthy participants. Statistical analysis was performed with two‐way ANOVA. Significant difference (*p* < 0.01) between: (*) AP vs. RP; (**) AP vs. HS; (***) RP vs. HS.

Abbreviations: AP, active pancolitis; HS, healthy subjects; RP, Remission pancolitis.

**FIGURE 2:**
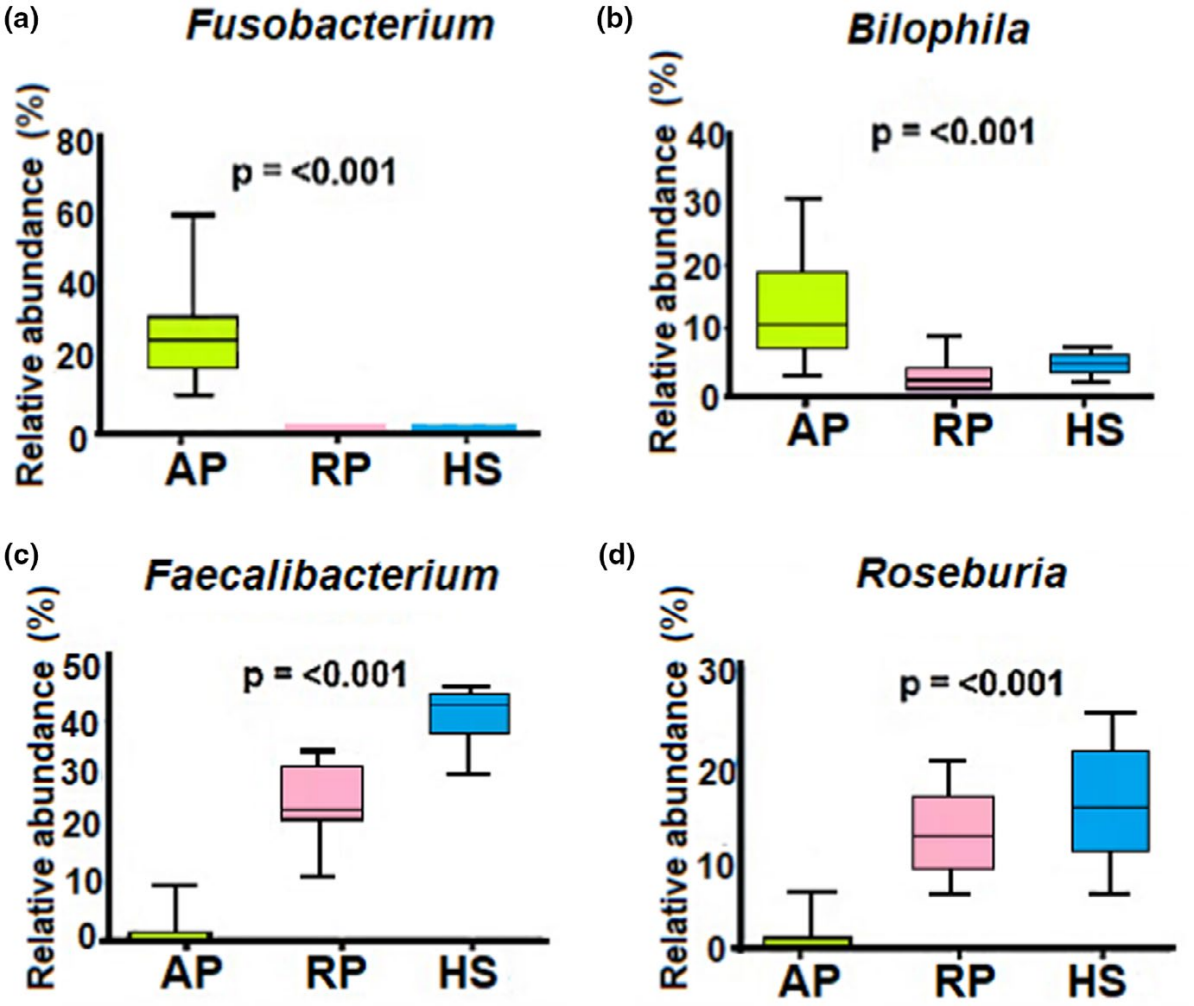
Abundance analyses. Whisker‐box plots comparing bacterial genera in the fecal microbiota of pancolitis, remission, and healthy participants. Only the four most relevant bacterial genera, according to abundant taxonomic composition, were analyzed: (a) *Fusobacterium*; (b) *Bilophila*; (c) *Faecalibacterium*, and (d) *Roseburia*. the *p*‐value indicates the statistical significance of two‐way ANOVA. Abbreviations: AP, active pancolitis; HS, healthy subjects; RP, Remission pancolitis

Interestingly, the relative abundance of the most frequent bacterial genus observed in active pancolitis was significantly different from those corresponding to remission pancolitis and healthy participants (Table 2; Figure 2).

**FIGURE 3:**
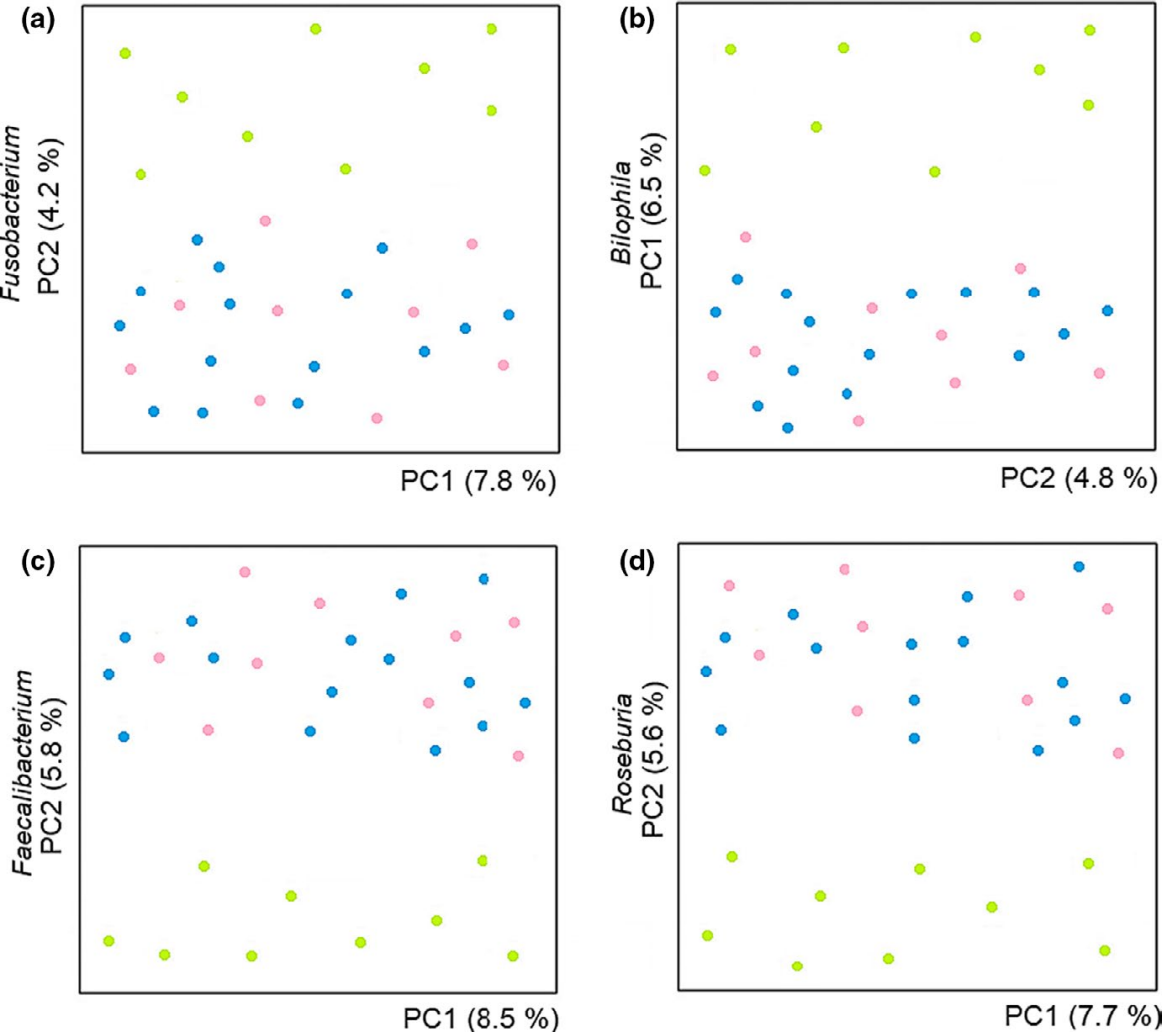
Gut microbiota abundance and UC severity marker. The plots show the clusters between the bacterial genera in the fecal microbiota with calprotectin, a biomarker of UC severity. The four most abundant bacterial genera, according to abundant taxonomic composition: (a) *Fusobacterium*; (b) *Bilophila*; (c) *Faecalibacterium*, and (d) *Roseburia*; were analyzed in the subgroups of active pancolitis (green), remission pancolitis (pink), and healthy participants (blue)

The structure of the most abundant microbial species was explored with the biomarker of UC severity, fecal calprotectin; where the clusters of the different phases are significantly separated based on fecal calprotectin (Figure 3).

**FIGURE 4:**
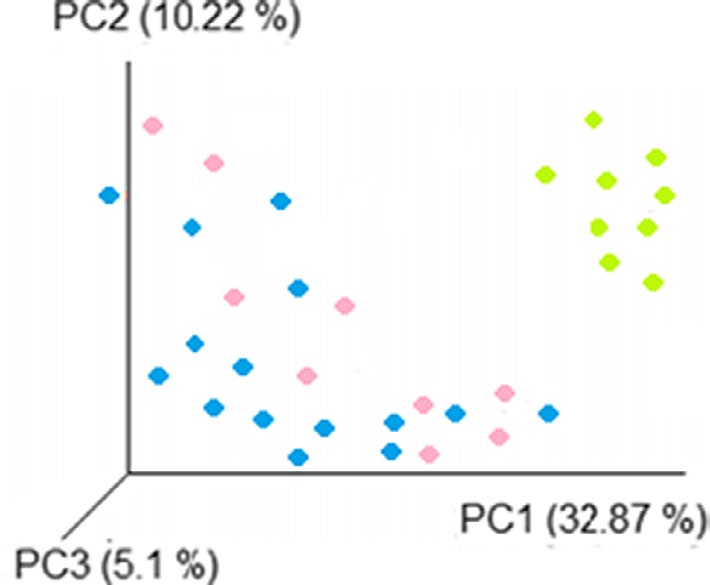
Principal component analysis. The overall structure of the fecal microbiota was plotted according to the different clinical scenarios (active pancolitis [green], remission pancolitis [pink], and healthy participants [blue]). Each data point represents an individual sample

The microbial community structure was investigated by using the principal component analysis, which allows splitting particular microbial communities according to their potential relation with clinical scenarios. Highly defined microbiota clusters were distributed according to clinical phases of pancolitis, where the distribution of active pancolitis clusters was significantly separated from those of remission pancolitis and healthy participants, showing these two last clusters a closer distribution between them (Figure 4).

**FIGURE 5:**
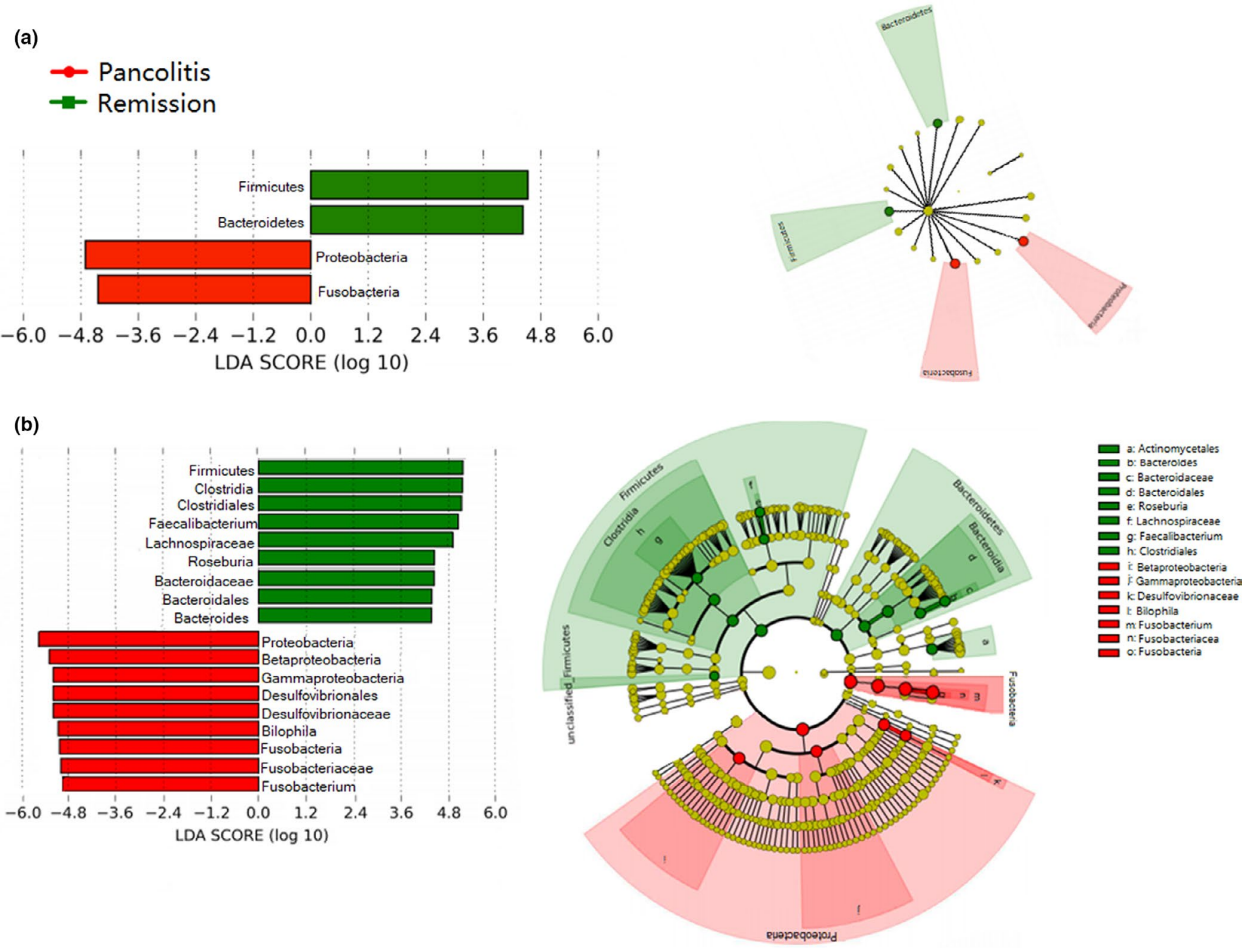
The linear discriminant analysis effect size (LEfSe) analysis of fecal microbiota with active pancolitis vs. remission pancolitis. (A) Bar graph showing LDA scores of phylum and cladogram generated by LEfSe indicating differences at phylum among active pancolitis and remission pancolitis. (b) Bar graph showing LDA scores of genus and cladogram generated by LEfSe indicating differences at genus among active pancolitis and remission pancolitis. Each successive circle represents a phylogenetic level. Regions in red indicate taxa enriched in active pancolitis, while regions in green indicate taxa enriched in remission pancolitis. Differing genera are listed on the right side of the cladogram

Linear discriminant analysis (LDA) effect size (LEfSe) was used to determine differentially abundant bacterial taxa between active and remission pancolitis. Patients with active pancolitis were related to the phylum *Proteobacteria* and *Fusobacteria*, while patients in remission pancolitis were related to the phylum *Firmicutes* and *Bacteroidetes* (α = 0.01, LDA score >3.0) (Figure 5; Appendix: Table A5).

Finally, we explored whether the relative abundance of the bacterial genus most frequently observed (cutoff values according to ROC analysis: active vs. remission pancolitis *Bilophila* 10%, *Faecalibacterium* 40%; pancolitis vs. healthy participants *Bilophila* 10%, *Fusobacterium* 10%) were related with active pancolitis. *Bilophila* and *Fusobacterium* showed an AUC of 0.917 and 0.988 for active vs. remission pancolitis, while similar AUCs were observed for active pancolitis vs. healthy participants, but not for remission pancolitis vs. healthy participants (Appendix: Table A6).

## 4 DISCUSSION

Our main finding was the significant differences of fecal microbiota composition from patients with active vs. remission pancolitis, with potential clinical application. Our study population was constituted of young aged patients with UC and severe stage of pancolitis. Scarce studies have explored gut microbiota in such population, probably due to the low prevalence of pancolitis between cases with UC. However, gut dysbiosis observed in patients with active vs. remission pancolitis in the present study is comparable with other reports (Alam et al., 2020; Danilova et al., 2019; Franzosa et al., 2019; Halfvarson et al., 2017; Imhann et al., 2018; Kumari et al., 2013; Sha et al., 2013). Our results were further validated by comparison with gut microbiota from healthy participant controls from a family who shares a similar diet and they are expected to exert a lower influence on the gut microbiota composition.

Our results showed an increased proportion of the phylum *Proteobacteria* and the genera *Fusobacterium* and *Bilophila* in active pancolitis, which was significantly different from the group of remission pancolitis and healthy participants, who shared a microbiota profile of higher proportion of phylum *Firmicutes*, and genera *Faecalibacterium* and *Roseburia* (Vester‐Andersen et al., 2019). These results are similar to those obtained by Franzosa et al., 2019, Kumari et al., 2013; Sha et al., 2013. Particularly, the findings of a reduced proportion of the genera *Faecalibacterium* *and* *Roseburia* in active pancolitis, and their restoration in remission pancolitis, has also been observed in previous reports (Khan et al., 2019; Man et al., 2011; Palmela et al., 2018; Vigsnæs et al., 2012). Such characterization is relevant due to scanty information regarding microbiota abundance in the remission phase of pancolitis, whereas consistent identification of specific genus in the remission phase may be useful to design more efficient therapeutic strategies, prompted to reduce UC severity. Interestingly, a particular bacterial composition like *Faecalibacterium* was shared by remission pancolitis and healthy participants. These bacteria have been reported to metabolize dietary components that promote colonic motility, maintain the intestinal immune system, and anti‐inflammatory properties (Dicks et al., 2018). Consistently, reduced abundance of these microorganisms has been associated with a higher rate of recurrence of UC (Alam et al., 2020; Al‐Bayati et al., 2018; Ferreira‐Halder et al., 2017; Kinross et al., 2011; Lopez‐Siles et al., 2017; Machiels et al., 2014) although increased levels of *Faecalibacterium* in stool samples have been associated with a lower activity index, supporting their role as potential biomarkers of disease severity and outcome, as suggested in other studies (Paramsothy et al., 2019; Wang et al., 2018).

Other findings were the higher abundance of the phylum *Proteobacteria*, and particularly the expansion of the genus *Bilophila* in active pancolitis. It is known that the relative abundance of *Bilophila* is promoted by diets enriched in saturated fats, which increase bacterial resistance to bile elimination. Furthermore, a change in the type of fat consumed affects the composition of gut microbiota, which may modify the onset and severity of UC (Devkota & Chang, 2015; Pittayanon et al., 2020; Torres et al., 2018). Dietary modifications involving excessive consumption of fried food, dairy products, and wheat flour are associated with the development of severe diarrhea in patients with active pancolitis (Keshteli et al., 2019). In the present study, we consider that there is no significant effect derived from the modification of the diet, since the population consumed a soft diet with abundant hydration; without a specific recommendation for dietary restrictions, even during active pancolitis.

Certain species of *Fusobacterium* show pro‐inflammatory, invasive, and adherent capacity to the intestinal mucosa, while the increased proportion of *Bilophila* in the gut promotes an immune response mediated by Th1, resulting in the development of colitis in experimental mice models (Bashir et al., 2016; Chen et al., 2020; Hirano et al., 2018; Liu et al., 2019; Ohkusa et al., 2002; Tahara et al., 2015; Wright et al., 2015). Although a direct pathophysiological mechanism is not possible to elucidate from the present study, we can propose that the relative abundance of some species is associated with the degree of inflammation and pancolitis, derived from the inverse relationship observed between the abundance of *Fecalibacterium* and *Roseburia* with calprotectin, a biomarker of severity of UC, which was consistent with a recent report (Björkqvist et al., 2019; Yu et al., 2019). Likewise, differences in bacterial richness, diversity, and dominance were highly related to the clinical scenarios studied. Remarkably, remission pancolitis and healthy participants showed the highest relative abundance of the phylum *Firmicutes*, which contributed to most of the bacterial diversity and richness (Björkqvist et al., 2019; Ganji‐Arjenaki & Rafieian‐Kopaei, 2018; Jandhyala et al., 2015). Further analyses of cluster distribution of bacterial communities showed differences in active pancolitis, as compared to remission pancolitis and healthy participants, which was consistent with previous studies showing a difference in the structure of microbiota between active pancolitis and healthy participants (Forbes et al., 2016; Havenaar, 2011; Louis & Flint, 2017).

Furthermore, studies characterizing gut microbiota composition and its modification during pancolitis are relevant, since (a) pancolitis provides a higher risk for colorectal cancer, whereas gut dysbiosis is thought to facilitate colorectal cancer development; (b) the study of gut microbial communities during clinical phases of pancolitis contributes to a better understanding of potential interactions with the host immune response; (c) characterization of a specific genus of gut microbial communities may own potential clinical application derived from their association with pancolitis or remission phases; and (d) specific microbial manipulation, concomitant to antibiotic use, is currently used as a therapeutic approach for UC (Alard et al., 2018; Devkota & Chang, 2015; Galazzo et al., 2019).

Finally, gut dysbiosis has been proposed as an important contributing factor to the increasing prevalence of pancolitis, with a potential role for the related clinical‐therapeutic phases (Halfvarson et al., 2017; Miyoshi et al., 2018; Petrof et al., 2013). Consistently, we found a significant ability of the genus *Bilophila* and *Fusobacterium* to selectively associate with cases of activity/remission pancolitis (Fukuda & Fujita, 2014; Guo et al., 2019).

To our knowledge, this is the first study that investigated the composition of fecal microbiota in Mexican patients with active and remission pancolitis. Our study faces some limitations. First, *16S* *rRNA* analysis provides the taxonomic composition of the microbes present in the community and does not provide an analysis of the role of the microbiota in the disease. Second, data analysis may show limitations regarding the specific characterization of microbiota composition as an isolated endpoint; however, we think that the analysis performed yields an adequate interpretation within a translational context, highlighting the role of microbiota diversity in the clinical phases of pancolitis. Third, larger sample size may be required to confirm our data and further research is required to better characterize the role of gut microbiota in patients with pancolitis.

Here, we provide a broad investigation of the fecal microbial community in Mexican patients presenting pancolitis. We demonstrate differences in the microbiota communities in patients with active pancolitis, remission pancolitis, and healthy participants. Selective association of gut dysbiosis with active/remission pancolitis may set the basis for further applications of non‐invasive methods, clinically useful for early identification of disease severity.

## CONFLICTS OF INTEREST

None declared.

## AUTHOR CONTRIBUTIONS

**Brenda Maldonado Arriaga:** Conceptualization (equal); Formal analysis (equal); Investigation (equal); Methodology (equal); Writing‐original draft (equal); Writing‐review & editing (equal). **Sergio Sandoval‐Jimenez:** Formal analysis (equal); Writing‐original draft (equal); Writing‐review & editing (equal). **Juan Rodriguez ‐Silverio:** Formal analysis (equal); Writing‐original draft (equal); Writing‐review & editing (equal). **Sofía Lizeth Alcaráz‐Estrada:** Formal analysis (equal); Writing‐original draft (equal); Writing‐review & editing (equal). **Tomás Cortés‐Espinosa:** Formal analysis (equal); Writing‐original draft (equal); Writing‐review & editing (equal). **Rebeca Pérez‐Cabeza de Vaca:** Formal analysis (equal); Writing‐original draft (equal); Writing‐review & editing (equal). **Cuauhtémoc Licona‐Cassani:** Data curation (equal); Formal analysis (equal); Writing‐original draft (equal); Writing‐review & editing (equal). **July Stephany Gámez‐Valdez:** Data curation (equal); Formal analysis (equal); Writing‐original draft (equal); Writing‐review & editing (equal). **Jonathan Shaw:** Formal analysis (equal); Investigation (equal); Writing‐original draft (equal); Writing‐review & editing (equal). **Paul Mondragón‐Terán:** Investigation (equal); Writing‐original draft (equal); Writing‐review & editing (equal). **Cecilia Hernández‐Cortez:** Writing‐original draft (equal); Writing‐review & editing (equal). **Juan Antonio Suárez‐Cuenca:** Conceptualization (equal); Formal analysis (equal); Investigation (equal); Validation (equal); Writing‐original draft (equal); Writing‐review & editing (equal). **GRACIELA CASTRO‐ESCARPULLI:** Conceptualization (equal); Formal analysis (equal); Investigation (equal); Supervision (equal); Writing‐original draft (equal); Writing‐review & editing (equal).

## ETHICS STATEMENT

The study was carried out according to the 1975 ethical guidelines of the Declaration of Helsinki. All participants provided written informed consent. The study was approved by the Local Committees of Research, Ethics in Research and Biosafety of the *Centro* *Médico* *Nacional* *‘20* *de* *Noviembre’* ISSSTE, Mexico City (Protocol ID No. 358.2017).

## Data Availability

Sequence data are available in the NCBI repository under BioProject accession number PRJNA596546: https://www.ncbi.nlm.nih.gov/bioproject/596546

## 1

**TABLE A1:** List of bacterial taxa with their OTUs ID in active pancolitis

No.	OTUs ID	*Bacteria*	No.	OTUs ID	*Bacteria*
1	925	*Brenneria*	49	471278	*Buttiauxella*
2	1006	*Curtobacterium*	50	479863	*Shimwellia*
3	1163	Microbacteriaceae	51	484145	Enterobacteriaceae
4	4981	*Rothia*	52	489853	*Cronobacter*
5	5294	Micrococcaceae	53	498961	*Klebsiella*
6	5605	Trueperella	54	501249	Enterobacteriaceae
7	6509	*Actinomycetaceae*	55	504890	*Phocoenobacter*
8	7967	*Actinomyces*	56	526378	Pasteurellaceae
9	8584	Actinomycetaceae	57	528975	Pasteurellaceae
10	9376	*Anaerofilum*	58	542569	*Mannheimia*
11	10096	Ruminococcaceae	59	554567	*Actinobacillus*
12	10256	*Sporosarcina*	60	561469	*Aggregatibacter*
13	10458	Planococcaceae	61	566893	Pasteurellaceae
14	10725	*Gemella*	62	576178	*Haemophilus*
15	11203	Bacillales	63	580361	Pasteurellaceae
16	14506	*Pediococcus*	64	603577	Fusobacteriaceae
17	14625	Lactobacillaceae	65	640658	Fusobacteriaceae
18	15213	*Tetragenococcus*	66	648973	*Ilyobacter*
19	16721	Enterococcaceae	67	689746	*Propionigenium*
20	19563	*Delftia*	68	704128	Fusobacteriaceae
21	125300	Comamonadaceae	69	728910	*Bacteroides*
22	128695	Burkholderiales	70	731897	Bacteroidaceae
23	132065	Neisseriaceae	71	735981	*Blautia*
24	136219	*Rhodospirillum*	72	765640	Lachnospiraceae
25	139865	Rhodospirillaceae	73	777038	*Aeromonas*
26	174563	Rhodospirillales	74	796328	Aeromonadaceae
27	176398	*Gemmiger*	75	857896	*Coprococcus*
28	182397	Hyphomicrobiaceae	76	871259	Lachnospiraceae
29	187569	Hyphomicrobiaceae	77	901257	Ruminococcaceae
30	195637	Rhizobiales	78	943078	*Ruminococcus*
31	198759	Campylobacterales	79	963365	Ruminococcaceae
32	201289	*Campylobacter*	80	1005973	*Pseudomonas*
33	206986	Campylobacteraceae	81	1019639	Pseudomonadaceae
34	217893	Campylobacterales	82	1025697	*Enterococcus*
35	241479	Pseudomonadales	83	1056986	Enterococcaceae
36	274893	Aeromonadaceae	84	1078963	Bacilli
37	289963	*Shewanella*	85	1086935	*Escherichia*
38	301329	Shewanellaceae	86	1087789	*Fusobacterium*
39	325698	Alteromonadales	87	1096348	Fusobacteriaceae
40	341449	Enterobacteriaceae	88	1098986	Fuobacteria
41	371893	*Yersinia*	89	1118963	*Bilophila*
42	378963	*Providencia*	90	1116035	Desulfovibrionaceae
43	390132	*Pantoea*	91	1131789	Desulfovibrionales
44	405698	Enterobacteriales	92	1135348	Deltaproteobacteria
45	424147	*Citrobacter*	93	1137986	Enterobacteriaceae
46	444893	*Raoultella*	94	1142963	Gammaproteobacteria
47	459993	*Kluyvera*	95	1145350	Enterobacteriaceae
48	461329	Enterobacteriaceae	96	1147789	Betaproteobacteria

**TABLE A2:** List of bacterial taxa with their OTUs ID in remission pancolitis

No.	OTUs ID	*Bacteria*	No.	OTUs ID	*Bacteria*
1	9012	*Peptococcus*	49	502589	*Pediococcus*
2	9378	*Trueperella*	50	527810	*Lactobacillus*
3	9646	Actinomycetaceae	51	530182	*Pilibacter*
4	9899	Peptostreptococcaceae	52	537891	Enterococcaceae
5	10463	*Dialister*	53	540128	Lactobacillales
6	10896	*Adlercreutzia*	54	562189	*Anaerococcus*
7	11302	*Bacteroides*	55	567812	*Peptoniphilus*
8	14567	Bacteroidaceae	56	572143	*Parvimonas*
9	14996	Clostridiales	57	577810	Clostridiales
10	15027	Ruminococcaceae	58	583071	*Pseudoramibacter*
11	15201	*Atopobium*	59	594777	*Eubacterium*
12	16457	*Lactobacillus*	60	617802	Eubacteriaceae
13	16830	*Collinsella*	61	631492	Clostridiales
14	18473	*Bradyrhizobium*	62	637490	*Peptococcus*
15	18990	Rikenellaceae	63	658714	Peptococcaceae
16	117963	Clostridiaceae	64	669748	*Acetanaerobacterium*
17	118634	*Bifidobacterium*	65	689816	*Ruminococcus*
18	119012	Bifidobacteriaceae	66	705933	*Butyricicoccus*
19	119986	Peptostreptococcaceae	67	729801	*Flavonifractor*
20	122479	*Catenibacterium*	68	742137	Clostridium IV
21	126239	Bacteroidales	69	775910	Ruminococcaceae
22	129301	Barnesiellaceae	70	778426	*Parasporobacterium*
23	134203	Erysipelotrichaceae	71	798763	Lachnospiraceae
24	137748	*Prevotella*	72	825479	*Dorea*
25	150193	*Phascolarctobacterium*	73	876900	*Coprococcus*
26	189760	*Parabacteroides*	74	889861	Lachnospiraceae
27	199127	*Porphyromonas*	75	918733	Ruminococcaceae
28	215079	*Odoribacter*	76	934759	*Peptostreptococcus*
29	215630	*Butyricimonas*	77	989744	Peptostreptococcaceae
30	217998	*Barnesiella*	78	1012989	*Anaerostipes*
31	260327	Porphyromonadaceae	79	1026597	Lachnospiraceae
32	269160	*Prevotella*	80	1044390	*Pediococcus*
33	285496	Prevotellaceae	81	1073619	Lactobacillaceae
34	290112	*Alistipes*	82	1079801	*Enterococcus*
35	301028	Rikenellaceae	83	1093607	*Bacteroides*
36	303241	*Staphylococcus*	84	1107218	Bacteroidaceae
37	339875	Staphylococcaceae	85	1128970	Bacteroidales
38	340178	Bacillales	86	1140126	Enterococcaceae
39	346920	*Aerococcus*	87	1149566	Clostridiales
40	375984	Aerococcaceae	88	1151490	Lachnospiraceae
41	379617	*Granulicatella*	89	1156301	*Blautia*
42	398863	Carnobacteriaceae	90	1189612	Ruminococcaceae
43	420159	*Weissella*	91	1211437	*Roseburia*
44	426713	Leuconostocaceae	92	1237019	Lachnospiraceae
45	456906	Enterococcaceae	93	1240789	Clostridiales
46	479802	*Streptococcus*	94	1246772	*Faecalibacterium*
47	481590	Streptococcaceae	95	1250116	Ruminococcaceae
48	499301	Lactobacillales	96	1257301	Clostridiales

**TABLE A3:** List of bacterial taxa with their OTUs ID in healthy participants

No.	OTUs ID	*Bacteria*	No.	OTUs ID	*Bacteria*
1	11456	*Rhodospirillum*	49	583010	*Dorea*
2	11895	*Duodenibacillus*	50	588322	*Roseburia*
3	11634	Lactobacillales	51	593701	Lachnospiraceae
4	12581	*Collinsella*	52	601086	*Faecalibacterium*
5	12988	*Atopobium*	53	605865	Clostridiales
6	16217	Ruminococcaceae	54	612789	*Bifidobacterium*
7	18759	Bacteroidales	55	645277	Lachnospiraceae
8	24663	*Enterococcus*	56	648712	*Blautia*
9	169384	Leuconostocaceae	57	657899	Ruminococcaceae
10	183691	Bacteroidaceae	58	678970	*Sutterella*
11	183968	Enterococcaceae	59	698782	Rikenellaceae
12	214569	*Streptococcus*	60	719017	*Prevotella*
13	221447	*Bacteroides*	61	733265	Lactobacillales
14	225896	Streptococcaceae	62	739100	*Staphylococcus*
15	237562	Enterococcaceae	63	753101	Staphylococcaceae
16	268741	*Porphyromonas*	64	772368	*Odoribacter*
17	271458	Ruminococcaceae	65	794890	Porphyromonadaceae
18	276398	*Phascolarctobacterium*	66	827745	Eubacteriaceae
19	281145	*Butyricimonas*	67	875214	*Eubacterium*
20	287482	*Parvimonas*	68	889803	Clostridiales
21	301189	*Pseudoramibacter*	69	914732	*Prevotella*
22	308756	Clostridiales	70	963365	Prevotellaceae
23	324470	*Peptococcus*	71	980217	*Butyricicoccus*
24	328621	Peptococcaceae	72	998510	Ruminococcaceae
25	331398	*Butyricicoccus*	73	1038960	*Clostridium*
26	335563	*Parabacteroides*	74	1041517	*Parasporobacterium*
27	346308	*Barnesiella*	75	1048796	*Dorea*
28	362391	*Flavonifractor*	76	1057893	Lachnospiraceae
29	367522	*Peptostreptococcus*	77	1096780	Clostridiales
30	371145	Peptostreptococcaceae	78	1101203	*Mobiluncus*
31	376598	*Acetanaerobacterium*	79	1106891	*Actinomyces*
32	379856	Lachnospiraceae	80	1110289	Actinomycetaceae
33	392350	*Pediococcus*	81	1115981	*Prevotella*
34	398571	*Anaerostipes*	82	1145780	*Dialister*
35	402265	*Enterococcus*	83	1148765	Clostridiales
36	428796	Lactobacillaceae	84	1150127	Lachnospiraceae
37	441278	Ruminococcaceae	85	1159812	*Bacteroides*
38	449127	Ruminococcaceae	86	1163970	Bacteroidales
39	451281	*Ruminococcus*	87	1167095	*Ruminococcus*
40	456580	Clostridiaceae	88	1208976	Bacteroidaceae
41	459102	*Bifidobacterium*	89	1220178	*Blautia*
42	467458	Bifidobacteriaceae	90	1227141	Ruminococcaceae
43	481097	Peptostreptococcaceae	91	1240139	*Roseburia*
44	489810	*Catenibacterium*	92	1247890	Lachnospiraceae
45	501433	Bacteroidales	93	1261372	*Faecalibacterium*
46	510129	*Bacteroides*	94	1266716	Ruminococcaceae
47	514780	Barnesiellaceae	95	1284820	Clostridiales
48	568894	Erysipelotrichaceae	96	1289362	Clostridiales

**TABLE A4:** Diversity Indexes for pancolitis phases and healthy participants

Diversity index	HS	AP	RP	*p*‐value
Observed	7122.82	2321.01	4138.14	0.001
Chao1	4215.58	1112.32	3241.56	0.001
Shannon	4.1	1.0	3.6	0.001
Simpson	0.48	1.2	0.52	0.001

*p*‐value indicates the statistical significance of 2‐way ANOVA.

Abbreviations: AP, active pancolitis; HS, healthy participants; RP Remission pancolitis.

**TABLE A5:** Linear discriminant analysis (LDA) effect size (LEfSe) analysis for active pancolitis and remission pancolitis

Taxa	Group	LDA score	*p*‐value
p_Proteobacteria;c_Betaproteobacteria	Active Pancolitis	4.8999	*0.042*
p_Proteobacteria;c_Gammaproteobacteria	Active Pancolitis	4.8963	*0.0210*
p_Proteobacteria;c_Deltaproteobacteria;o_Desulfovibrionales	Active Pancolitis	4.8912	0.0160
p_Proteobacteria;c_Deltaproteobacteria;o_Desulfovibrionales;f_Desulfovibrionaceae	Active Pancolitis	4.8836	0.0309
p_Proteobacteria;c_Deltaproteobacteria;o_Desulfovibrionales;f_Desulfovibrionaceae;g_*Bilophila*	Active Pancolitis	4.8792	0.0436
p_Fusobacteria;c_Fusobacteria	Active Pancolitis	4.8569	0.0122
p_Fusobacteria;c_Fusobacteria;o_Fusobacteriales;f_Fusobacteriaceae	Active Pancolitis	4.8498	0.0132
p_Fusobacteria;c_Fusobacteria;o_Fusobacteriales;f_Fusobacteriaceae;g_*Fusobacterium*	Active Pancolitis	4.8475	0.0145
p_Firmicutes;c_Clostridia	Remission Pancolitis	4.8961	0.0394
p_Firmicutes;c_Clostridia;o_Clostridiales	Remission Pancolitis	4.8926	0.0058
p_Firmicutes;c_Clostridia;o_Clostridiales;f_Ruminococcaceae;g_*Faecalibacterium*	Remission Pancolitis	4.8912	0.0132
p_Firmicutes;c_Clostridia;o_Clostridiales;f_Lachnospiraceae	Remission Pancolitis	4.8859	0.0246
p_Firmicutes;c_Clostridia;o_Clostridiales;f_Lachnospiraceae;g_*Roseburia*	Remission Pancolitis	4.6898	0.0322
p_Bacteroidetes;c_Bacteroidia;o_Bacteroidales;f_Bacteroidaceae	Remission Pancolitis	4.6889	0.0125
p_Bacteroidetes;c_Bacteroidia;o_Bacteroidales	Remission Pancolitis	4.6885	0.0203
p_Bacteroidetes;c_Bacteroidia;o_Bacteroidales;f_Bacteroidaceae;g_*Bacteroides*	Remission Pancolitis	4.6880	0.0209

The threshold on the logarithmic LDA score for discriminative features was set to 2.0. The name of a higher taxon level was added before its taxon abbreviation. “p”, phylum; “c”, class; “o”, order; “f”, family; “g”, genus. “LDA” Linear discriminant analysis. *p* < 0.05 are considered statistically significant.

**TABLE A6:** Diagnostic performance of *Bilophila*, *Fusobacterium*, *Faecalibacterium*, and *Roseburia* in discriminating Pancolitis‐related conditions

	AUC	Se (%)	Sp (%)	PPV (%)	NPV (%)
AP vs. HS					
***Bilophila***	**0.917**	80	100	100	83
***Fusobacterium***	**0.955**	90	100	100	90
*Faecalibacterium*	0.222	0	80	0	44
*Roseburia*	0.237	0	90	0	47
AP vs. RP					
***Bilophila***	**0.917**	80	100	100	83
***Fusobacterium***	**0.955**	90	100	100	90
*Faecalibacterium*	0.206	10	10	10	10
*Roseburia*	0.237	0	90	0	47
RP vs. HS					
*Bilophila*	0.000	N/A	100	N/A	50
*Fusobacterium*	0.000	N/A	100	N/A	50
*Faecalibacterium*	0.237	90	0	47	0
*Roseburia*	0.400	40	60	50	50

Cutoffs. HS vs. AP discrimination: Bilophila (10%), Fusobacterium (10%), Faecalibacterium (45%), Roseburia (20%). AP vs RP discrimination: Bilophila (10%), Faecalibacterium (40%), Roseburia (20%). HS vs RP discrimination: Bilophila (5%), Fusobacterium (5%), Faecalibacterium (20%), Roseburia (10%).

Abbreviatures: AP, Active Pancolitis; AUC, Area Under the Curve; HS, Healthy Subjects; N/A, non‐applicable; NPV, Negative Predictive Value; PPV, Positive Predictive Value; RP, Remission Pancolitis; Se, Sensitivity; Sp, Specificity. Bold letters and values indicates the most abundant and have statistical significance.
